# Pharmacotherapy of psychiatric inpatients with mental and behavioral disorders caused by sedatives or hypnotics (F13): Results from an observational pharmacovigilance program between 2000 and 2017

**DOI:** 10.1186/s13722-025-00579-6

**Published:** 2025-06-20

**Authors:** Philipp Pauwels, Beatrice Haack, Sermin Toto, Johanna Seifert, Renate Grohmann, Martin Heinze, Oliver Zolk, Phileas Johannes Proskynitopoulos, Michael Schneider, Timo Greiner

**Affiliations:** 1 Brandenburg Medical School, University Clinic for Psychiatry and Psychotherapy, Immanuel Klinik Rüdersdorf, 15562 Rüdersdorf, Germany; 2 Department of Psychiatry, Social Psychiatry and Psychotherapy, Hannover Medical School, 30625 Hannover, Germany; 3 Department of Psychiatry and Psychotherapy, LMU University Hospital, LMU Munich, Nussbaumstr. 7, 80336 Munich, Germany; 4 Institute of Clinical Pharmacology of the Brandenburg Medical School, Immanuel Klinik Rüdersdorf, 15562 Rüdersdorf, Germany

**Keywords:** Sedatives, Hypnotics, Anxiolytics, Psychotropic drugs, Tranquilizing drugs, AMSP, Pharmacovigilance, F13

## Abstract

**Background:**

The treatment of choice for substance use disorder (SUD) caused by sedatives, hypnotics, and anxiolytics (SHA) is to slowly taper the dose of the implicated substance to the point of abstinence, thereby minimizing withdrawal symptoms. Concomitant pharmacotherapy may be indicated to manage excessive withdrawal or rebound symptoms. This study investigated the use of psychotropic drugs for the treatment of SHA-dependent SUD patients in Germany.

**Methods:**

Data were obtained from the German *Drug Safety Program in Psychiatry* (“Arzneimittelsicherheit in der Psychiatrie”; AMSP) from 2000 to 2017. SHA SUD was classified using the 10th Edition of the International Classification of Disease (ICD-10).

**Results:**

The present study included 1,015 patients with a primary diagnosis of mental and behavioral disorders due to use of sedatives or hypnotics (F13.1-F13.9). The most common comorbid psychiatric diagnoses were additional SUDs (F1, *n* = 279), especially alcohol use disorder (F10; *n* = 124), and mood disorders (F3; *n* = 201). A total of 95.6% of patients received one or more psychotropic drugs, most commonly antidepressant drugs (63.1% of patients), tranquillizing drugs (55.5%), and antipsychotic drugs (46.7%). The most common combination was an antidepressant drug and a tranquillizing drug (33.0%). Overall, psychotropic drugs with sedating properties (e.g., mirtazapine, quetiapine, doxepin, and trimipramine) were preferred. An increase in use over the 17-year observation period was observed for tranquillizing drugs and, most pronounced, for antipsychotic drugs.

**Conclusion:**

We found high rates of non-SHA drugs among patients treated for SHA-SUD. The prevalent use of psychotropic drugs with strong sedating properties indicates a symptom-oriented treatment approach, which is often “off-label” but may be clinically necessary. Main limitation is the cross-sectional design of the AMSP databank. Therefore, we cannot provide any follow up data on the patient collective especially regarding outcome.

**Supplementary Information:**

The online version contains supplementary material available at 10.1186/s13722-025-00579-6.

## Background

Sedatives, hypnotics, and anxiolytics (SHAs) are among the drugs with the highest potential for dependence and misuse. Within the broad category of SHAs, benzodiazepines and Z-drugs are the most implicated in SHA-related substance use disorders (SUD) [1, 2], conceivably in relation to their action at inhibitory GABA-receptors in the brain. Individual benzodiazepines and Z-drugs differ in their SUD-inducing potential due to their pharmacodynamic properties [3]. Off-label and long-term use of SHAs are particularly problematic [4, 5]. SHAs may be indicated– at least temporarily– for comorbidities such as insomnia, schizophrenia spectrum disorders, and depression, which, however, can contribute to the development of SHA-SUDs [6–11]. Co-occurring SUDs of other substances are common comorbidities of a primary SHA-SUD [12].

The rate of SHA use is estimated at 5% in Germany and 9.7% in Switzerland, whereas the prevalence of SHA-SUD among persons aged 16–64 years is approximately 1.4% in Germany [13, 14]. As older females seem at particular risk for SHA-SUDs [15–17], some prevalence reports may underestimate the issue, due to their focus on those aged less than 65 years. A study analyzing long-term benzodiazepine utilization (i.e., > 6 months) among older patients in Germany reported that benzodiazepine utilization increased with age, from 12.3% among patients aged 65–70 years to 31.6% among patients aged > 90 years [18]. The same study did not detect significantly higher rates for females, which is, however, a known risk factor for SHA-SUD [18, 19].

The treatment of SHA-SUDs can be challenging, partly because of the frequent comorbidity with other SUDs. The main goal of treatment is to reach a state of complete abstinence of the implicated SHA, which can be achieved by slowly titrating down the dose, ideally avoiding severe withdrawal symptoms [2, 15]. However, withdrawal symptoms often occur despite careful dose titration; typical psychological and physical symptoms include insomnia (71–74%), anxiety (49–56%), muscle spasms or pain (49%), mood swings/testiness (49%), and tremor (38–46%). Seizures are among the most severe complications but fortunately rarely occur in controlled inpatient settings [20–22]. Due to the potentially life-threatening nature of SHA withdrawal, patients usually require inpatient care for dose titration. Treatment of SHA-SUD should be individualized including nonpharmacological interventions, such as cognitive behavioral therapy, dose reduction of the respective SHAs, and pharmacotherapy that excludes SHAs [15, 23].

Overall, evidence supporting the use of specific drugs such as pregabalin, paroxetine, or tricyclic antidepressants for withdrawal treatment is weak [15, 24]. In addition to the paucity of robust studies on non-SHA pharmacological strategies, real-world data from clinical practice are also lacking. To the best of our knowledge, there are currently no published studies providing information regarding pharmacological treatment strategies for inpatients with SHA-SUD.

To provide novel information within this under-researched area, we analyzed prescription data from a pharmacovigilance database for the period 2000–2017 for inpatients who were diagnosed with mental and behavioral disorders caused by SHAs, according to the 10th revision of the International Statistical Classification of Disease and Related Health Problems (ICD-10, F13 category). The ICD-10 criteria for SUD consists of somatic, psychological, and behavioral symptoms which largely overlap with the criteria in the Diagnostic and Statistical Manual of Mental Disorders, Fifth Edition (DSM-5). Our analysis focuses on psychotropic drug use patterns and associated time trends for different drugs and drug groups. Additionally, we stratified our analyses by sex and the presence or absence of psychiatric comorbidities.

## Methods

The data within the present study were collected by the German Drug Safety Program in Psychiatry (Arzneimittelsicherheit in der Psychiatrie; AMSP), an ongoing multicenter national drug surveillance program which has been collecting data on pharmacotherapy and severe adverse drug reactions (ADRs) in psychiatric inpatients in German-speaking countries since 1993. A list of all participating centers is available online (www.amsp.de). AMSP was initiated to improve the safety of pharmacological treatment in psychiatry. In 2017, 52 psychiatric hospitals in Germany, Austria, and Switzerland participated in the AMSP program. AMSP methods have been described in detail elsewhere [25]. In brief, data on ADRs and on drug use are stored in separate databanks. Participating hospitals gather anonymous information on psychotropic and nonpsychotropic drug use for all hospitalized psychiatric patients on two reference days each year. Apart from drug use data and their daily doses, we documented the primary psychiatric diagnosis, sex, and age. As of 2007, secondary and tertiary psychiatric diagnoses were also gathered. Psychiatric diagnoses were recorded by the treating psychiatrist according to the ICD-10 (from 2000 to present). Data are stored in an anonymized data bank designed to ensure that subjects cannot be backtracked. Thus, it is impossible by design and not intended to gather follow-up data of the included patients on those two reference days each year. However, a patient might appear several times if hospitalized on multiple reference days. The present evaluations based on AMSP data were approved by the Ethics Committee of the University of Munich and the Ethics Committee of the Hannover Medical School (Nr. 8100_BO_S_2018) and adhere to the Declaration of Helsinki and its later amendments.

Our analysis uses data from the AMSP database from 2000 to 2017, including hospitals in Germany (*n* = 78), Austria (*n* = 18), and Switzerland (*n* = 22). All patients included in this study had a primary diagnosis (i.e., main reason for the current hospitalization) of mental and behavioral disorders due to the use of sedatives or hypnotics (ICD-10 diagnosis category F13). Patients with acute intoxication (F13.0) were excluded from the analysis, as that condition is not necessarily indicative of an actual SUD but merely describes a transient state. Demographic and drug prescription data were analyzed for the study population.

Psychotropic drugs were categorized into the following main drug groups: antidepressant drugs, antipsychotic drugs, tranquilizing drugs, hypnotic drugs, and antiepileptic drugs. Antipsychotic drugs were further categorized as first-generation antipsychotic drugs (FGAs) and further into high and low potency and second-generation antipsychotic drugs (SGAs). Subgroups of antidepressant drugs were classified as selective serotonin reuptake inhibitors (SSRIs), selective serotonin noradrenaline reuptake inhibitors (SSNRIs), noradrenergic and specific serotonergic antidepressants (NaSSAs), tricyclic antidepressants (TCAs), and “other antidepressants” (e.g., substances not classified in the previous subgroups). Supplemental Table 1 shows drugs used in ≥ 2% of the study population within the previously named categories.

All statistical analyses were performed using Excel. We calculated relative ratios and used descriptive statistics (arithmetic means ± standard deviation (SD), median, range). The percentages presented were calculated in relation to the entire study population. Where applicable, data were analyzed with Student’s t-test for means or the χ²-test for significant differences in proportions. The level of significance was set at *p* < 0.05. Data was also stratified by diagnosis, age, and sex.

Dose equivalents were calculated based on *defined daily doses* (DDDs) published by the Collaborative Center for Drug Statistics Methodology of the World Health Organization (WHO). DDDs have several benefits: (1) DDDs are available for most drugs, (2) they constitute an international measure, and (3) low- and high-dose spectrums can indicate the indications for which they may have been prescribed [26].

To analyze time trends, we clustered data into groups containing roughly the same number of patients. The first time period consisted of five years (2000–2004, *n* = 176), the second consisted of four years (2005–2008, *n* = 223), and the last three consisted of three years each (2009–2011, *n* = 210; 2012–2014, *n* = 207; 2015–2017, *n* = 199). For the time period 2007–2017, the databank contains information regarding psychiatric diagnoses in addition to the primary diagnosis.

## Results

From 2000 to 2017, the AMSP database comprised 1,058 patients with a primary diagnosis of mental and behavioral disorders due to use of sedatives or hypnotics (F13.X). Data from 43 patients (4.1%) with acute intoxication (F13.0) were removed, resulting in a final study population of 1,015 patients. The vast majority (827 patients, i.e., 82%) had a primary diagnosis of dependency syndrome (F13.2), whereas 81 (8%) were diagnosed with withdrawal syndrome (F13.3), and 57 (6%) were diagnosed with harmful use (F13.1). All other subdiagnoses (i.e., F13.4–13.9) constituted < 5% of patients (*n* = 43) (Table 1). Relative to the entire population of the AMSP database, which included a total of 153,088 patients during the analyzed time period, the prevalence of SHA-SUD as a primary diagnosis among psychiatric inpatients was 0.66%.

Patients with SHA SUD were predominantly female (*n* = 631, 62.2%), who were also older than their male counterparts (58.6 ± 14.9 years vs. 51.4 ± 15.1 years; t(1,013) = 7.4282, *p* < 0.001). The age range of both sexes was similar (females 18–90 years vs. males 15–92 years). The distribution according to age groups is shown in Table 1.

**Table 1:** Study population by age group and relative occurrence of primary diagnosis by subdiagnosis (total *n* = 1015)

Age groups	all ages *n* (% of 1015)	≤ 30 *n* (% of 78)	31–60 *n* (% of 615)	≥ 61 *n* (% of 322)
Female	631 (62.2)	35 (44.9)	348 (56.6)	248 (77.0)
Male	384 (37.8)	43 (45.1)	267 (43.4)	74 (23.0)
**Primary Diagnosis**				
**F13.1**	68 (6.7)	9 (11.5)	36 (5.9)	23 (7.2)
Female	35 (3.4)	4 (5.1)	14 (2.3)	17 (5.3)
Male	33 (3.3)	5 (6.4)	22 (3.6)	6 (1.9)
**F13.2**	827 (81.5)	60 (76.9)	526 (85.5)	241 (74.8)
Female	511 (50.3)	27 (34.6)	297 (48.3)	187 (58.1)
Male	316 (31.1)	33 (42.3)	229 (37.2)	54 (16.8)
**F13.3**	81 (8.0)	3 (3.8)	39 (6.3)	39 (12.1)
Female	59 (5.8)	1 (1.3)	29 (4.7)	29 (9.0)
Male	22 (2.2)	2 (2.6)	10 (1.6)	10 (3.1)
**F13.4**	25 (2.5)	-	7 (1.1)	18 (5.6)
Female	19 (1.9)	-	5 (0.8)	14 (4.3)
Male	6 (0.6)	-	2 (0.3)	4 (1.2)
F13.5– F13.9*	14 (1.4)	6 (7.7)	7 (1.1)	1 (0.3)
Female	7 (0.7)	3 (3.8)	3 (0.5)	1 (0.3)
Male	7 (0.7)	3 (3.8)	4 (0.7)	-

Main group F13 = Mental and behavioral disorders due to sedatives or hypnotics| Subdiagnoses: 1 = harmful use; 2 = dependence syndrome; 3 = withdrawal state; 4 = withdrawal state with delirium; 5 = psychotic disorder; 6 = amnesic syndrome; 7 = residual and late-onset psychotic disorder; 9 = unspecified mental and behavioral disorder

*= F13.8 was not present in our population

### Analysis of comorbid mental disorders

Table 2 provides a detailed overview of comorbid mental disorders. For the time period of 2007–2017, additional diagnoses were reported (*n* = 726), there of 530 patients (73%) had a comorbidity. Among these, 279 (38.4%) patients had additional mental and behavioral disorders due to any substance abuse (F1); most often due to alcohol (F10, 17.1%). Patients with a comorbid diagnosis of alcohol use disorder were predominantly male (58.1%) and had a mean (SD) age of 51.7 ± 11.8 years. The second most common additional diagnosis was mood disorder (F3). The 201 of the 726 patients with additional mood disorders also presented demographic differences; these patients were more often female (69.2%) and had a mean age of 56.3 ± 14.4 years.

**Table 2:** Relative occurrence of additional psychiatric diagnoses for the period 2007–2017 (*n* = 726)

ICD-10-Code	Additional diagnoses
	n (% of 726)
No additional diagnosis	196 (27.0)
F0 Organic, including symptomatic, mental disorders	22 (3.0)
F1 Mental and behavioral disorders due to psychoactive substance use	279 (38.4)
F10 due to use of alcohol	124 (17.1)
F11 due to use of opioids	70 (9.6)
F12 due to use of cannabinoids	22 (3.0)
** F13 due to use of sedatives or hypnotics**	73 (10.1)
F14 due to use of cocaine	13 (1.8)
F15 due to use of other stimulants, including caffeine	12 (1.7)
F16 due to use of hallucinogens	2 (0.3)
F17 due to use of tobacco	25 (3.4)
F18 due to use of volatile solvents	-
F19 due to multiple drug use and use of other psychoactive substances	20 (2.8)
F2 Schizophrenia, schizotypal and delusional disorders	29 (4.0)
F3 Mood [affective] disorders	201 (27.7)
F4 Neurotic, stress-related and somatoform disorders	114 (15.7)
F5 Behavioral syndromes associated with physiological disturbances and physical factors	3 (0.4)
F6 Disorders of adult personality and behavior	47 (6.5)
F7 Mental retardation	3 (0.4)
F8 Disorders of psychological development	1 (0.1)
F9 Unspecified mental disorder	5 (0.7)
G0 Inflammatory diseases of the central nervous system	1 (0.1)

### Psychotropic drug use

Overall, 98.5% of all patients utilized some type of drug (*n* = 1,000) (Table 3); 95.6% used at least one psychotropic drug (*n* = 970), and 27.8% received > 3 psychotropic drugs, resulting in a mean (SD) of 2.8 ± 1.6 psychotropic drugs per patient. Over time, the mean number of psychotropic drugs per patient increased significantly from the first time period (2000–2004) to the last time period (2015–2017) (2.4 ± 1.4 vs. 2.8 ± 1.7; t(373)=-2.653, *p* = 0.008).

The mean number of psychotropic drugs per patient was significantly higher for patients with an additional diagnosis of mood disorders than for patients without any additional diagnosis or within the SHA-SUD spectrum (3.1 ± 1.5 vs.2.9 ± 1.4); t(926)=-2,219, *p* = 0.027). For patients with a comorbid alcohol use disorder, the difference was not statistically significant (2.9 ± 1.4 vs. 2.6 ± 1.5; t(848) = 1.759, *p* = 0.08).

Table 3 shows the use of psychotropic drugs. Antidepressants (61.3%) and tranquilizing drugs (55.5%) were the most frequently used, followed by antipsychotic and antiepileptic drugs (46.7% and 37.6%, respectively). Hypnotic drugs (14.8%) were used more rarely. Among antidepressant drugs, NaSSAs (mainly mirtazapine) were the most used subgroup (21.3%), followed by SSRIs, TCAs, and SSNRIs (18.7, 17.6, and 12.8%, respectively). “Other antidepressants” were less frequently prescribed (5.9%), mostly trazodone and agomelatine. Among antipsychotic drugs, the most frequently used were SGAs (27.2%), most often quetiapine (53.6% of all SGAs) and various low-potency FGAs (24.9%). Antidepressant drugs, low-potency FGAs, and tranquilizing drugs were more commonly used in females. The most prescribed hypnotic drugs were zolpidem, zopiclone, and valerian (41.3, 28.7, and 16.0% of all hypnotic drugs, respectively). Diazepam, lorazepam, and oxazepam were the most commonly used tranquilizing drugs (45.8, 27.4, and 18.8% of all tranquilizing drugs, respectively). The most commonly used antiepileptic drugs were carbamazepine, pregabalin, and clonazepam (24.9%, 21.5%, and 16.2% of all antiepileptic drugs, respectively). Within this group, pregabalin and clonazepam were prescribed more to males than to females (Table 3).

**Table 3:** Use of psychotropic drug groups and individual psychotropic drugs* overall and by sex

	All patients	Female patients	Male patients	Difference between male and female patient (χ²| p)	
	*n* (% of 1,015)	*n* (% of 631)	*n* (% of 384)		
Any medication	1,000 (98.5)	621 (98.4)	379 (98.7)	0.131	0.717
Any psychotropic drug	970 (95.6)	606 (96.0)	364 (94.8)	0.875	0.35
Antidepressant drugs	622 (61.3)	**408 (64.7)**	**214 (55.7)**	**8.023**	**0.005**
SSRIs	190 (18.7)	128 (20.3)	62 (16.1)	2.688	0.101
- Citalopram	52 (5.1)	38 (6.0)	14 (3.6)	2.773	0.096
- Sertalin	43 (4.2)	23 (3.6)	20 (5.2)	1.438	0.23
- Escitalopram	57 (5.6)	40 (6.3)	17 (4.4)	1.647	0.199
NaSSAs	216 (21.3)	144 (22.8)	72 (18.8)	2.361	0.124
- Mirtazapine	213 (21.0)	141 (22.3)	72 (18.8)	1.861	0.172
SSNRIs	130 (12.8)	80 (12.7)	50 (13.0)	0.025	0.874
- Venlafaxine	82 (8.1)	50 (7.9)	32 (8.3)	0.054	0.816
- Duloxetine	47 (4.6)	29 (4.6)	18 (4.7)	0.005	0.946
TCAs	179 (17.6)	115 (18.2)	64 (16.7)	0.4	0.528
- Doxepin	73 (7.2)	41 (6.5)	32 (8.3)	1.205	0.272
- Trimipramin	70 (6.9)	45 (7.1)	25 (6.5)	0.143	0.705
“Other antidepressants”	60 (5.9)	**45 (7.1)**	**15 (3.9)**	**4.465**	**0.035**
- Trazodone	38 (3.7)	28 (4.4)	10 (2.6)	2.226	0.136
Antipsychotic drugs	474 (46.7)	309 (49.0)	165 (43.0)	3.454	0.063
SGAs	276 (27.2)	173 (27.4)	103 (26.8)	0.043	0.837
- Quetiapine	148 (14.6)	93 (14.7)	55 (14.3)	0.033	0.856
- Olanzapine	55 (5.4)	38 (6.0)	17 (4.4)	1.185	0.276
- Risperidone	48 (4.7)	26 (4.1)	22 (5.7)	1.371	0.242
Low-potency FGAs	253 (24.9)	**173 (27.4)**	**80 (20.8)**	**5.529**	**0.019**
- Pipamperone	69 (6.8)	**51 (8.1)**	**18 (4.7)**	**4.343**	**0.037**
- Prothipendyl	50 (4.9)	36 (6.0)	17 (4.4)	2.162	0.141
- Chlorprothixene	49 (4.8)	26 (5.7)	23 (6.0)	1.815	0.178
- Promethazine	47 (4.7)	35 (5.5)	12 (3.1)	3.17	0.075
- Melperon	37 (3.6)	26 (4.1)	11 (2.9)	1.072	0.301
High-potency FGAs	41 (4.0)	27 (4.3)	14 (3.6)	0.247	0.619
Hypnotic drugs	150 (14.8)	100 (15.8)	50 (13.0)	1.515	0.218
- Zolpidem	62 (6.1)	45 (7.1)	17 (4.4)	3.044	0.081
- Zopiclone	43 (4.2)	27 (4.3)	16 (4.2)	0.007	0.931
- Valerian*	24 (2.4)	**17 (2.7)**	**7 (1.8)**	**9.273**	**0.002**
Antiepileptic drugs	382 (37.6)	**210 (33.3)**	**172 (44.8)**	**13.477**	**0**
- Carbamazepine	95 (9.4)	62 (9.8)	33 (8.6)	0.427	0.513
- Pregabalin	82 (8.1)	47 (7.4)	35 (9.1)	0.892	0.345
- Clonazepam	62 (6.1)	**27 (4.3)**	**35 (9.1)**	**9.733**	**0.002**
- Gabapentin	61 (6.0)	**23 (3.6)**	**38 (9.9)**	**16.513**,	**0**
- Valproate	61 (6.0)	35 (5.5)	26 (6.8)	0.633	0.426
- Levetiracetam	37 (3.6)	**16 (2.5)**	**21 (5.5)**	**5.847**	**0.016**
- Oxcarbazepine	31 (3.1)	**13 (2.1)**	**18 (4.7)**	**5.565**	**0.018**
Tranquilizing drugs	563 (55.5)	**367 (58.2)**	**196 (51.0)**	**4.899**	**0.027**
- Diazepam	258 (25.4)	171 (27.1)	87 (22.7)	2.486	0.115
- Lorazepam	154 (15.2)	102 (18.1)	52 (13.5)	1.276	0.259
- Oxazepam	106 (10.4)	65 (11.5)	41 (10.7)	0.036	0.849

SSRI: selective serotonin reuptake inhibitor; SSNRI: selective serotonin-norepinephrine reuptake inhibitor; TCA: tricyclic antidepressant; NaSSA: noradrenergic and specific serotonergic antidepressant; FGA: “first-generation antipsychotic drug”; SGA: “second-generation antipsychotic drug”

* Only drugs prescribed to ≥ 2.5% of patients are shown except for valerian for better displaying the hypnotic drug group

χ²: Chi-square distribution; p: p value

### Psychotropic drug use in patients with comorbid mental disorders

We repeated the previous analysis for the time period 2007–2017, for which data regarding additional diagnoses were available, and then compared psychotropic drug use in patients without comorbid mental disorders or only additional SHA-SUD diagnosis to that in patients with additional psychiatric diagnoses other than a SHA-SUD. Overall, utilization patterns were similar between these subgroups (Supplemental Table 2). However, upon further subgroup examination, we found some relevant differences for two comorbidities: patients with comorbid mood disorders more often received antidepressant (83.4 vs. 57.7%, *p* < 0.001) and antipsychotic drugs (51.8 vs. 48.2%, *p* = 0.017) than patients without any comorbidity or only in the realm of a SHA-SUD (F13). In contrast, patients with additional alcohol use disorder diagnosis had lower utilization rates for antipsychotic (39.5%) and antidepressant drugs (55.6%), but this difference was not statistically significant.

### Psychotropic drugs and dosages

Diazepam was the most frequently used drug (26.6%), followed by mirtazapine (22.0%), lorazepam (15.9%), quetiapine (15.3%) and oxazepam (10.9%). The use of zolpidem (6.4%) and zopiclone (4.3%) was much lower (Table 4).

High median DDDs were recorded for venlafaxine (1.5), escitalopram (1.5), and zolpidem (1.1), whereas clonazepam (0.2), pipamperone (0.2), chlorprothixene (0.2), prothipendyl (0.3), and quetiapine (0.4) were used at lower median DDDs. The median diazepam dosage was 1.0 (Table 4). Pipamperone was more frequently used in female patients, whereas clonazepam and gabapentin were more frequently used to treat males. The median DDDs of males were markedly greater for escitalopram and citalopram (1.5 vs. 1.0 and 1.8 vs. 1.0, respectively; Supplemental Table 3).

**Table 4:** Most common drugs* with daily defined dose (DDD) and absolute dosages

Drug	n (% of 1,015)	DDD	Absolute Dosage
		Median (Q1; Q3)	Median (Q1; Q3)
Diazepam	258 (26.6)	1.0 (0.4; 2.0)	10 (4; 20)
Mirtazapine	213 (22.0)	1.0 (0.5; 1.5)	30 (15; 45)
Lorazepam	154 (15.9)	0.6 (0.4; 1.2)	1.5 (1; 3)
Quetiapine	148 (15.3)	0.4 (0.2; 0.8)	150 (75; 300)
Oxazepam	106 (10.9)	0.6 (0.4; 1.0)	30 (20; 50)
Carbamazepine	95 (9.8)	0.6 (0.4; 0.6)	600 (400; 600)
Pregabalin	82 (8.5)	1.0 (0.5; 1.3)	300 (150; 375)
Venlafaxine	82 (8.5)	1.5 (1.1; 2.3)	150 (106.25; 225)
Doxepin	73 (7.5)	1.0 (0.8; 1.3)	100 (75; 125)
Trimipramine	70 (7.2)	0.7 (0.3; 1.0)	100 (50; 150)
Pipamperone	69 (7.1)	0.2 (0.2; 0.3)	40 (40; 60)
Clonazepam	62 (6.4)	0.2 (0.1; 0.4)	1.88 (1; 3.5)
Zolpidem	62 (6.4)	1.1 (1.0; 2.0)	11.1 (10; 20)
Gabapentin	61 (6.3)	0.7 (0.4; 1.1)	1200 (800; 2000)
Valproate	61 (6.3)	0.7 (0.4; 0.9)	1000 (600; 1300)
Escitalopram	57 (5.9)	1.5 (1.0; 2.0)	15 (10; 20)
Olanzapine	55 (5.7)	1.0 (0.5; 1.6)	10 (5; 16.25)
Citalopram	52 (5.4)	1.0 (1.0; 2.0)	20 (20; 40)
Prothipendyl	50 (5.2)	0.3 (0.3; 0.5)	80 (80; 115)
Chlorprothixene	49 (5.1)	0.2 (0.2; 0.4)	65 (50; 105)

* Only drugs prescribed to ≥ 5% of patients are shown; Q1: first quartile; Q3: third quartile

### Use of combinations of psychotropic drugs

Concomitant use of an antidepressant and tranquilizing drug was most common and observed in 33.0% (*n* = 335) of all 1015 inpatients, followed by the combination of an antidepressant and an antipsychotic drug in 29.8% (*n* = 302) and an antipsychotic in combination with a tranquilizing drug in 26.8% (*n* = 272) of patients. A total of 9.7% of patients received more than one sedative hypnotic. A total of 5.6% of patients concomitantly received a Z-drug and a benzodiazepine, among which zolpidem and lorazepam were the most common combination.

### Time trends of psychotropic drug use

**Fig. 1:**
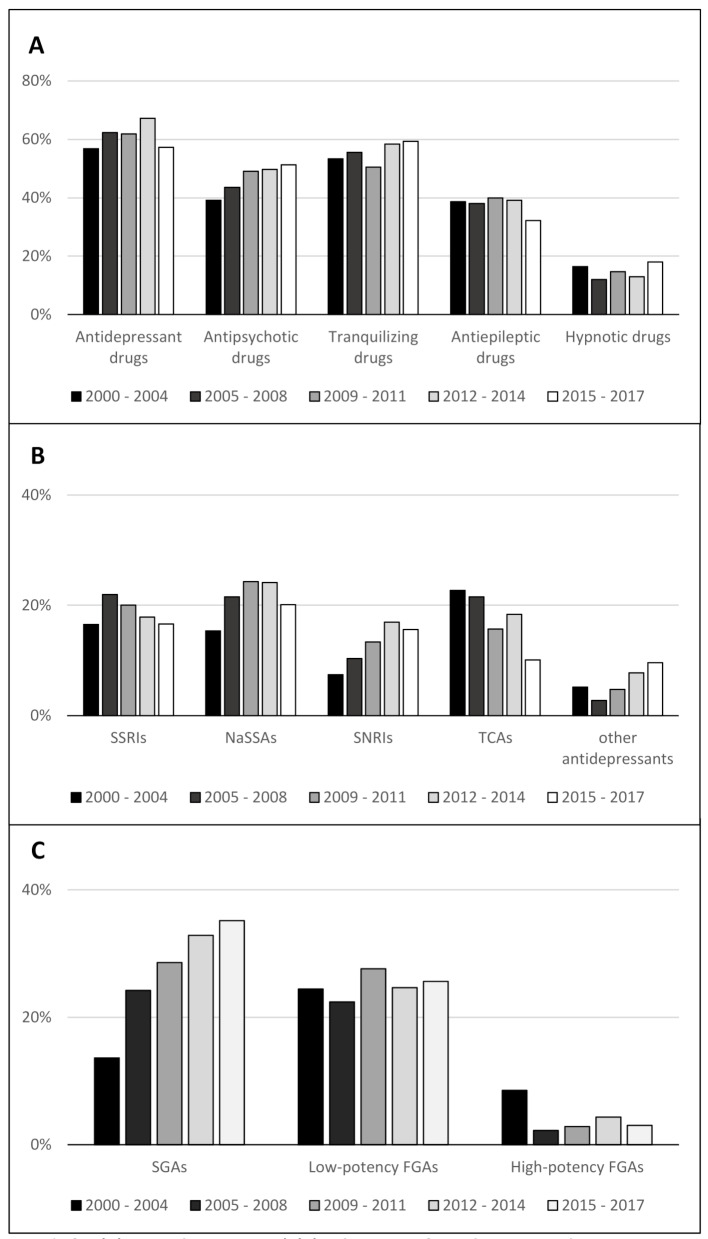
Time trends for **(A)** main drug groups| **(B)** subgroups of antidepressant drugs SSRIs: serotonin reuptake inhibitors; NaSSAs: noradrenergic and specific serotonin reuptake inhibitors; SSNRIs: selective serotonin noradrenaline reuptake inhibitors; TCAs: tricyclic antidepressants and other antidepressants| **(C)** subgroups of antipsychotic drugs SGAs: second-generation antipsychotic drugs; FGA: first-generation antipsychotic Drug use as a percentage of the study population during the displayed time period

Within the different time periods, the utilization rates of antidepressant drugs ranged between 57% and 67%. The use of antipsychotic drugs increased from 39% in the first period to 51% (*p* = 0.190) in the last period. Tranquilizing drug use also increased over time from 53 to 59% (*p* = 0.251), whereas hypnotic drug use fluctuated slightly between 12% and 18% over time (Fig. 1A). All results were statistically not significant.

Among antidepressant drugs, the use of SSNRIs (7.4 to 15.6%, *p* = 0.014) and other antidepressants (5.1 to 9.5%, *p* = 0.103) increased over time. The use of TCAs decreased from 22.7 to 10.1% (*p* = 0.001) from the first to the last period, whereas the use of SSRIs decreased after a peak of 22% in the second period to 16.6% (*p* = 0.978) in the final period (Fig. 1B). Among antipsychotic drugs, the use of SGAs increased steeply from 13.6 to 35.2% (*p* < 0.001) over the five periods. The use of low-potency FGAs remained stable at approximately 25%, whereas the use of high-potency FGAs decreased over time from 8.5 to 3.0% (*p* = 0.021) (Fig. 1C).

We repeated the analysis for our female and male cohort. Due to small groups sizes, we still saw similar trends, but none were statistically significant.

## Discussion

We analyzed real-world patterns of psychotropic drug use in psychiatric inpatients with mental and behavioral disorders due to SHAs in German-speaking countries between 2000 and 2017. To date, no other studies have focused on the psychotropic drug use patterns of inpatients with this primary diagnosis.

We found a high rate of psychotropic drug use (95.6%) in this patient group, comparable to rates for patients with major depressive disorder (MDD; 95.8%) and schizophrenia (98.5%) within the AMSP program [9, 27]. The mean number of psychotropic drugs per patient (2.8 ± 1.6) was comparable to that for general psychiatric inpatients within the AMSP database (2.6–2.8; [28]), suggesting that (psycho)polypharmacy is common in this subgroup of psychiatric inpatients. Polypharmacy increases the risk of drug-drug as well as drug-disease interactions potentially worsening the patient’s condition [29]. The number of drugs is, however, greater than that for psychiatric outpatients attending a tertiary care hospital (2.3 ± 0.90 [30] and 1.8 ± 1.0 [31]). These discrepancies might be due to the presumably more stable condition of outpatients. In general, additional psychiatric comorbidities had no significant effect on psychotropic drug use, as shown in Supplemental Table 2. Considering the risks of (psycho)polypharmacy, concomitant psychiatric medication without proper indication should be seen critical. A careful approach to medication schemes is essential to optimize benefits while minimizing harm in this vulnerable group [29, 32].

There is often a comorbid association between alcohol use disorder and SHA-SUD [16, 33]. Accordingly, SHA-SUD with an additional alcohol use disorder was the most common combination of two SUDs in our sample. This association might reflect the convergence of pharmacological effects on the GABAergic system in the brain, increasing the risk of self-administering benzodiazepines as a means to overcome alcohol withdrawal [12, 34, 35]. However, iatrogenic exposure to benzodiazepines as part of alcohol withdrawal treatment might increase the risk of developing SHA-SUD [36]. Here, we found a considerably high clonazepam and gabapentin use. While there is broad appreciation of the risks posed by benzodiazepines, recent evidence emphasizes the abuse and toxicity of gabapentin, along with its use in conjunction with alcohol [37, 38].

Our finding that mood disorder was the second most common comorbidity suggests that our collective is susceptible to mood disorders. Indeed, there is commonly a co-occurrence of SUDs and mood disorders [39]. Moreover, patients undergoing treatment for mood disorders often receive SHAs for anxiety and/or insomnia, potentially increasing the risk of SHA-SUD development in this population, especially when SHAs are given for a prolonged period. SHAs may also be utilized not only to manage illness-emergent suicidality but also antidepressant drug-induced suicidality. This rare ADR requires immediate attention and benzodiazepines may provide fast relief [40]. Furthermore, SHAs are used to mitigate the delayed onset of action of antidepressant drugs. All these are indications for the short-term use of SHAs. The findings of a Cochrane review underscore no long-term benefit from concomitant use of antidepressant drugs and SHAs despite the rapid alleviation of insomnia and anxiety in the early phase of MDD or in the case of an ADR during antidepressant drug treatment [10, 41]. In addition, Ogawa et al. (2019) found that the frequency of reporting one or more ADRs was slightly higher in patients treated with an antidepressant drug and benzodiazepines (RR 1.12, 95% CI 1.01–1.23) [10]. We must also consider that SHAs can treat symptoms occurring during withdrawal from an antidepressant drug, which in recent years has been recognized to affect more than half of all patients treated with antidepressant drugs [42, 43]. Overall, any benefits gained from the use of SHAs in this patient subgroup should be carefully weighed against the potential harm, with careful management aiming to prevent SHA misuse [7].

More than 50% of patients in the present study received tranquilizing drugs. Compared with other collectives within the AMSP program, this utilization is as anticipated to be significantly greater than among patients with alcohol dependence (24.3%; unpublished observations of our workgroup, in preparation by Haak et al.), posttraumatic stress disorder (PTSD) (29.3%; [44]), MDD (29.9%; [27]), and schizophrenia (32.0%; [9]). However, considering that withdrawal from tranquilizing drugs is a main reason for the hospitalization of patients with a primary diagnosis of SHA-SUD, one might expect higher tranquilizing drug utilization rates, as controlled dose reduction is a main treatment strategy. Using reference day data only, AMSP data is lacking information about the status of the course of treatment.

The combination of a Z-drug and a benzodiazepine is apparently not uncommon in clinical practice, even though guidelines do not recommend the combination of two SHAs [15, 45]. Both sedatives have similar effects on the GABA receptor subtype A and a similar side effect profile, despite their differing structures. There is no clinical evidence that Z-drugs are less addictive [15]. Concomitant prescription of a Z-drug and a benzodiazepine might be justified when switching from Z-drugs to long-acting benzodiazepines such as diazepam, which is recommended by UK guidelines because of the greater control of dose tapering with drugs having a prolonged plasma half-life [45]. The German guidelines leave drug switching to the doctor’s discretion based on their assessment of the condition and needs of the individual patient [15]. Logistical reasons (e.g., drug availability, standardized dosage schema, drug formulation) may make it necessary to switch drugs [46].

Interestingly, valerian with longstanding traditional use for the treatment of insomnia, was the third most prescribed hypnotic drug in the collective. Although a meta-analysis suggested a certain publication bias favoring its efficacy [47], it has little toxicity and no known addictive potential. Valerian use is not reported in other AMSP studies; therefore, we are unable to make comparisons with other patient groups within the AMSP database [7].

The utilization rate of antidepressant drugs within the subgroup of patients with a comorbid diagnosis of mood disorder is comparable to that of inpatients with a primary diagnosis of MDD. However, we also found high utilization rates of antidepressant and antipsychotic drugs among patients without comorbid mental disorders, which suggests that these psychotropic drugs were used to target specific symptoms (i.e., agitation). Overall, we observed frequent and increasing use of drugs, such as quetiapine and mirtazapine, with sedative and sleep-inducing properties—a trend also observed in other studies, especially concerning quetiapine. Among antidepressant drugs, we observed a shift from a predominant utilization of SSRIs and TCAs at the beginning of the study period to SSNRIs (mostly venlafaxine) and NASSAs (mostly mirtazapine), as well as to “other antidepressants”. This shift seems to be a common pattern in the psychiatric inpatient setting [27, 48, 49]. Compared with “older” antidepressant drugs such as TCAs, “newer” antidepressant drugs (e.g., SSNRIs, SSRIs) are praised for their greater neuroreceptor and transporter selectivity and their lower risk of ADRs, especially in older patients [49].

Among antipsychotic drugs, quetiapine was the most utilized and the third most prescribed drug overall within our study population and showed a nearly fivefold increase in utilization from 2000 to 2017. Other studies within [28, 44] and separate from [50] the AMSP-Project have made similar observations. Quetiapine is presumably a popular choice due to its sedative properties and its relatively favorable profile of ADRs, especially with respect to the lower risk of extrapyramidal symptoms in comparison to other antipsychotic drugs [51]. The present finding of a DDD of 0.52 suggests mainly low-dose utilization of quetiapine, most likely for the indication of insomnia (i.e., off-label use). Although quetiapine is widely used, it is an antipsychotic drug with a certain risk for severe ADRs, even when it is used at low dosages, such as cardiovascular events, neutropenia, and metabolic syndrome [51–53]. Additionally, quetiapine on its own has the potential for misuse, especially in patients with a history of addiction. Off-label use of quetiapine and its use in conjunction with psychotropic polypharmacy also pose a risk for misuse [54, 55]. Both conditions are also common in our study population.

We found that low-potency FGAs, as well as potent sedative antidepressant drugs such as doxepin and trimipramine, were among the more commonly used drugs in the study population. Like quetiapine, these drugs are most likely used off-label, again indicating a symptom-driven treatment approach in which these drugs are exploited for their sedative properties. While guidelines do not recommend the use of specific drugs for SUD patients, they do generally recommend placing a treatment focus on reducing major withdrawal symptoms such as anxiety, sleep disturbances, and agitation [15, 45].

TCA utilization in our population (17.6%) was notably higher compared to AMSP studies of patients with alcohol use disorder (1.6%), PTSD (14.2%), and MDD (13.7%) [27, 44, 56]. The previously mentioned Cochrane review concluded that it remains uncertain whether TCAs facilitate the discontinuation of benzodiazepines or reduce withdrawal symptoms, which calls their use into question. However, the TCA doxepin is a very potent H_1_ antihistaminergic sedative, which might underly its higher representation in our collective, in keeping with our other observations of the high use of drugs with sedating properties.

The overall utilization (37.6%) of antiepileptic drugs in our population is remarkably greater than that reported by Haak et al. in patients with alcohol use disorder within the AMSP program (23%) [56]. However, there is no clear recommendation for the use of antiepileptic drugs in SHA-SUD. This may reflect a type of substitution function in SUD and appears to be given to alleviate symptoms of withdrawal, given the GABA-mimetic properties attributed to pregabalin and gabapentin [38, 57]. Even more so, practitioners should be aware of the SUD potential of gabapentinoids, especially in patients with a history of SUD [57, 58].

We found a higher prevalence of female patients suffering from SHA-SUDs who were also older than male patients in our patient collective. These observations are consistent with those of Panes et al. and Olfson et al., who reported that an age exceeding 44 years and female sex were risk factors for the abuse of benzodiazepines [59, 60]. This could be related to the higher prevalences of anxiety disorders and insomnia, especially in older females [61, 62]. In comparison, patients with alcohol dependence were predominantly male (69.4%) in a comparable study in which AMSP data were used [56]. Within our collective, we detected relevant sex differences regarding the underlying comorbidities. Male patients with a diagnosis of SHA-SUDs were more likely to suffer from comorbid alcohol use disorders. Similarly, females in our collective had a higher prevalence of comorbid mood disorders.

### Limitations

Because data for this study derive from routine clinical data, there is incomplete documentation of relevant aspects such as interfering somatic diagnoses and comorbid psychiatric diagnoses extending over the entire 2000–2017 time period. Data regarding the clinical aspects of treatment (e.g., course of clinical symptoms, occurrence of ADRs, nonpharmacological treatment) or treatment outcome (i.e., clinical response to drug treatment) are not available. Thus, we cannot address correlation between clinical presentation and use patterns. Heterogenous institutional treatment practices or patient populations cannot be ruled out. Furthermore, we are unable to differentiate between low-dose and high-dose benzodiazepine dependency. Switching between drugs using cross-tapering strategies may have led to overestimations of drug use and the extent of polypharmacy. As in any study with a cross-sectional design, we cannot exclude the possibility that any observed changes were due to demographic shifts within the patient collective.

## Conclusion

Compared with male patients, female patients had a greater prevalence of SHA-SUD and were, on average, older. High rates of non-SHA drug use among patients with SHA-SUD are common and not sufficiently explained by the presence of comorbidities. The frequent use of psychotropic drugs with strong sedating properties is indicative of a symptom-oriented treatment approach, which may qualify as being “off-label”, despite being clinically necessary. In addition, the data also highlights that off label use seems to be common in SHA-SUD patients as various psychotropic drugs are used even without corresponding comorbidities present. Unexpectedly, real-world high use of antiepileptic drugs seemed to deviate from guideline recommendations, without clear evidence for efficacy in the context of SHA-SUD. Overall, the present results lead us to recommend continuing the monitoring of prescription patterns in SHA-SUD patients, and we see a need for expansion of the AMSP program to include more clinical sites; multicentric in-depth investigations could reveal the rationales and benefits of off-label drug utilization in this patient collective.

## Electronic supplementary material

Below is the link to the electronic supplementary material.

Supplementary Material 1

## Data Availability

No datasets were generated or analysed during the current study.

## Abbreviations

SUD: Substance use disorder
SHA: Sedatives, hypnotics, and anxiolytics
AMSP: Arzneimittelsicherheit in der Psychiatrie
ICD: International Classification of Diseases
DSM-V: Diagnostic and Statistical Manual of Mental Disorders, Fifth Edition
ADR: Adverse drug reactions
FGA: First-generation antipsychotic drug
SGA: Second-generation antipsychotic drug
SSRI: Selective serotonin reuptake inhibitor
SSNRI: Selective serotonin noradrenaline reuptake inhibitors
NaSSA: Noradrenergic and specific serotonergic antidepressant drug
SD: Standard deviation
DDD: Defined daily dose
WHO: World Health Organization
PTSD: Posttraumatic stress disorder
MDD: Major depressive disorder
