# Supplementary material for: Pharmacotherapy of psychiatric inpatients with mental and behavioral disorders caused by sedatives or hypnotics (F13): Results from an observational pharmacovigilance program between 2000 and 2017
Source: Addict Sci Clin Pract. 2025 Jun 20;20:51. doi: 10.1186/s13722-025-00579-6 (PMC12180187; doi:10.1186/s13722-025-00579-6)
Supplement: Supplementary file 1 — Supplementary Material 1 [file 13722_2025_579_MOESM1_ESM.docx]

**Supplement**

Supplemental Table 1. Classification of psychotropic drugs relevant to this study

| **Psychotropic drug group** | | **Subgroup** | **Individual drugs** |
| --- | --- | --- | --- |
| antidepressant drugs | | *SSRI* | escitalopram, citalopram, sertraline* |
|  |  | *SSNRI* | venlafaxine, duloxetine* |
|  |  | *TCA* | doxepin, trimipramine |
|  |  | *NaSSA* | mirtazapine |
|  |  | *other ADD* | trazodone* |
| antipsychotic drugs | FGA | lp FGA | pipamperone, prothipendyl, chlorprothixene*, promethazine*, melperone* |
|  |  | hp FGA | haloperidol** |
|  | SGA |  | quetiapine, olanzapine, risperidone*, aripiprazole** |
| hypnotic drugs | | Z-drug | zolpidem, zopiclone* |
|  |  |  | valerian ** |
| tranquilizing drugs | | benzodiazepine | diazepam, lorazepam, oxazepam |
| antiepileptic drugs | |  | carbamazepine, pregabalin, clonazepam, gabapentin, valproic acid, levetiracetam*, oxcarbazepine* |

*Supplemental Table 1. Classification of psychotropic drugs relevant to this study*

*SSRI: selective serotonin reuptake inhibitor; SSNRI: selective serotonin-norepinephrine reuptake inhibitor; TCA: tricyclic antidepressant; NaSSA: noradrenergic and specific serotonergic antidepressant; FGA: “first-generation antipsychotic drug”; lp: low potency; hp: high potency; SGA: “second-generation antipsychotic drug”*

**only drugs used in the treatment of ≥ 2.5% of patients are listed*

***because of very low overall utilization in these drug groups, drugs used in the treatment of ≥ 2.0% of patients are listed*

Supplemental Table 2. Use of psychotropic drug groups in patients with and without any additional psychiatric diagnoses other than F13 from 2007 - 2017 (total n=726)

*Supplemental Table 2. Use of psychotropic drug groups in patients with and without any additional psychiatric diagnoses other than F13 from 2007-2017 (total n=726)*

*F13 only*= Patients without any additional psychiatric diagnoses or only one or more additional psychiatric diagnoses within the SHA-SUD (F13) realm*

*SSRI: selective serotonin reuptake inhibitor; SSNRI: selective serotonin-norepinephrine reuptake inhibitor; TCA: tricyclic antidepressant; NaSSA: noradrenergic and specific serotonergic antidepressant; FGA: “first-generation antipsychotic drug”; SGA: “second-generation antipsychotic drug”*

*χ²: Chi-square distribution; p: p-value*

|  | F13 only*  *n* (% of 222) | F13 with additional F.- diagnoses  *n* (% of 504) | Difference between with and without additional F.- diagnoses groups (χ², p) |
| --- | --- | --- | --- |
| Any drug | 221(99.5) | 498 (98.8) | 0.884, 0.347 |
| Any psychotropic drug | 210 (95.0) | 480 (95.2) | 0.135, 0.713 |
| Antidepressant drugs  SSRIs  NaSSAs  SSNRIs  TCAs  “other antidepressants” | 128 (57.7)  40 (18.0)  55 (24.8)  31 (14.0)  30 (13.5)  9 (4.1) | 317 (62.9)  93 (29.3)  108 (34.1)  75 (23.7)  82 (29.3)  37 (11.7) | 1.783, 0.182  0.019, 0.889  0.991, 0.319  0.104, 0.747  0.897, 0.343  2.806, 0.094 |
| Antipsychotic drugs  SGAs  Low-potency FGAs  High-potency FGAs | 107 (48.2)  68 (30.6)  50 (22.5)  11 (5.0) | 223 (43.6)  158 (31.3)  134 (26.6)  13 (2.6) | 0.159, 0.691  0.037, 0.847  1.346, 0.246  2.721, 0.099 |
| Hypnotic drugs | 36 (16.2) | 75 (14.9) | 0.212, 0.645 |
| Antiepileptic drugs | 84 (37.8) | 197 (39.1) | 0.101, 0.750 |
| Tranquilizing drugs | 128 (57.7) | 279 (55.4) | 0.331, 0.565 |

| Drug | Female patients | | Male patients | | Difference between male and female patient  (χ², p) |
| --- | --- | --- | --- | --- | --- |
|  | *n* (% of 631) | DDD (median Q1 and Q3) | *n* (% of 384) | DDD (Mean ± SD) |  |
| Diazepam  Mirtazapine  Lorazepam  Quetiapine  Oxazepam  Carbamazepine  Venlafaxine  Pregabalin  Doxepin  Trimipramine  **Pipamperone**  **Clonazepam**  Zolpidem  **Gabapentin**  Valproate  Escitalopram  Olanzapine  Citalopram  Prothipendyl  Chlorprothixene | 171 (27.1)  141 (22.3)  102 (16.2)  93(14.7)  65 (10.3)  62 (9.8)  50 (7.9)  47 (7.4)  41 (6.5)  45 (7.1)  **51 (8.1)**  **27 (4.3)**  45 (7.1)  **23 (3.6)**  35 (5.5)  40 (6.3)  38 (6.0)  38 (6.0)  36 (5.7)  26 (4.1) | 1.33 ± 1.38  1.10 ± 0.57  0.91 ± 0.93  0.52 ± 0.47  0.92 ± 1.15  0.51 ± 0.25  0.84 ± 0.48  1.72 ± 0.98  1,09 ± 0,64  0.71 ± 0.37  0.24 ± 0.18  0.56 ± 1.43  1.63 ± 0.99  0.79 ± 0.55  0.65 ± 0.31  1.41 ± 0.67  1.10 ± 0.80  1.39 ± 0.77  0.38 ± 0.21  0.29 ± 0.17 | 87 (22.7)  72 (18.8)  52 (13.5)  55 (14.3)  41 (10.7)  33 (8.6)  32 (8.3)  35 (9.1)  32 (8.3)  25 (6.5)  **18 (4.7)**  **35 (9.1)**  17 (4.4)  **38 (9.9)**  26 (6.8)  17 (4.4)  17 (4.4)  14 (3.6)  14 (3.6)  23 (6.0) | 1.66 ± 2.45  1.07 ± 0.63  1.06 ± 1.01  0.73 ± 0.75  0.94 ± 0.86  0.60 ± 0.15  1.11 ± 0.65  1.76 ± 0.74  1.08 ± 0.62  0.74 ± 0.59  0.43 ± 0.34  0.49 ± 1.22  1.76 ± 1.49  0.83 ± 0.46  0.79 ± 0.43  1.45 ± 0.67  1.41 ± 0.84  1.48 ± 0.79  0.49 ± 0.30  0.31 ± 0.30 | 2.486, 0.115  1.861, 0.172  1.276, 0.259  0.03, 0.864  0.036, 0.849  0.427, 0.513  0.054, 0.816  0.892, 0.345  1.205, 0.272  0.143, 0.705  **4.343, 0.0372**  **9.733, 0.002**  3.044, 0.081  **16.513, <0.001**  0.633, 0.426  1.647, 0.199  1.185, 0.276  2.773, 0.096  2.162, 0.141  1.815, 0.178 |

Supplemental Table 3. Most common drugs* by sex with daily defined dose (DDD)

*Supplemental Table 3. Most common drugs* by sex with daily defined dose (DDD)*

** Only drugs prescribed to ≥ 5% of patients are shown. SD: standard deviation; χ²: chi-square distribution; p: p value*
